# Identification of a 20-Gene Expression-Based Risk Score as a Predictor of Clinical Outcome in Chronic Lymphocytic Leukemia Patients

**DOI:** 10.1155/2014/423174

**Published:** 2014-05-05

**Authors:** Elias Bou Samra, Bernard Klein, Thérèse Commes, Jérôme Moreaux

**Affiliations:** ^1^INSERM, U1040, F-34197, Institute of Research in Biotherapy, CHU Montpellier, 80 Avenue Augustin Fliche, 34285 Montpellier Cedex, France; ^2^CHU Montpellier, Institute of Research in Biotherapy, Montpellier, 80 Avenue Augustin Fliche, 34285 Montpellier, France; ^3^Université Montpellier 1, UFR Médecine, Montpellier, 80 Avenue Augustin Fliche, 34285 Montpellier, France; ^4^Institut de Biologie Computationnelle, Université Montpellier 2, 2 Place Eugène Bataillon, 34095 Montpellier Cedex 5, France

## Abstract

Despite the improvement in treatment options, chronic lymphocytic leukemia (CLL) remains an incurable disease and patients show a heterogeneous clinical course requiring therapy for many of them. In the current work, we have built a 20-gene expression (GE)-based risk score predictive for patients overall survival and improving risk classification using microarray gene expression data. GE-based risk score allowed identifying a high-risk group associated with a significant shorter overall survival (OS) and time to treatment (TTT) (*P* ≤ .01), comprising 19.6% and 13.6% of the patients in two independent cohorts. GE-based risk score, and *NRIP1* and *TCF7* gene expression remained independent prognostic factors using multivariate Cox analyses and combination of GE-based risk score together with *NRIP1* and *TCF7* gene expression enabled the identification of three clinically distinct groups of CLL patients. Therefore, this GE-based risk score represents a powerful tool for risk stratification and outcome prediction of CLL patients and could thus be used to guide clinical and therapeutic decisions prospectively.

## 1. Introduction

Chronic lymphocytic leukemia (CLL), the most common leukemia in the western countries, is characterized by the clonal proliferation and accumulation of neoplastic B lymphocytes in the blood, bone marrow, lymph nodes, and spleen. CLL shows a heterogeneous clinical course, with many patients having an indolent disease while others suffering from rapid disease progression and are in need of early treatment [1]. Clinical staging systems based on physical examination and routine laboratory tests are the first basis for assessing different prognostic subgroups in patients with CLL [1]. However, these staging systems have a limited capacity to predict clinical outcome at an early stage of the disease and do not predict the likelihood of response to treatment in an individual with advanced disease [2].

Several biomarkers have been identified out as prognostic factors in CLL. These include somatic hypermutations in the rearranged variable regions of the immunoglobulin heavy chain (*IgVH*) genes, which involve around 30–40% of patients. Patients with unmutated* IgVH* genes had a significantly shorter median overall survival (OS) than those with mutated ones [3].* IgVH* mutation status, along with deletions at 11q22-q23 (11q-) and/or 17p13 (17p-), has been identified as independent prognostic factors in CLL patients [4, 5].

Meanwhile, with the advent of microarray technology and gene expression profiling (GEP) analyses, additional markers have been investigated for their potential prognostic impact in CLL. Of these,* LPL* (Lipoprotein lipase) [6],* ZAP70* (zeta-associated protein 70) [7],* CLLU1 *(Chronic lymphocytic leukemia up-regulated 1) [8], and* TCL1A *(T-cell leukemia/lymphoma 1A) [9] have been demonstrated to be predictive for clinical outcome. Expression of microRNAs (e.g., miR-29c and miR-223) could be also of prognostic significance in CLL [10]. These markers combined with others were used to develop multigene expression-based prognostic scores. In 2006, Zucchetto et al. constructed a scoring system based on six surface expression molecules [11]. In a study by Rodríguez et al. [12], a predictor model based on the expression of seven genes allowed the characterization of three groups of patients with distinct OS and treatment-free survival (TFS), both in two independent cohorts of patients. In 2010, Kienle et al. identified a four-gene combination, based on* ZAP70*,* TCF7 *(Transcription factor 7),* DMD *(Dystrophin), and* ATM *(Ataxia telangiectasia mutated) expression, as a predictor of* IgVH* mutation status in 88% of cases [13]. Stamatopoulos et al. developed a qPCR score, based on the expression of three markers (*ZAP70*,* LPL* and miR-29c), that was able to significantly predict OS and TFS by dividing patients into three groups [14]. More recently, Herold et al. developed an eight-gene expression-based risk score which showed additional prognostic value for OS and TFS compared with the established genetic markers and Binet staging [15].

We report here the design of a GE-based risk score, involving 20 genes, whose value is strongly prognostic in 2 independent cohorts of CLL patients.

## 2. Methods

### 2.1. Patients

Gene expression microarray data from three independent cohorts of patients diagnosed with CLL were used. Publicly available gene expression data from 2 cohorts with newly diagnosed CLL patients were used to construct GE-based risk score [15]. The first cohort, used as the training cohort, comprised 107 patients, and the second one as the validation cohort comprised 44 patients [15]. Peripheral blood or bone marrow samples were analyzed by Affymetrix oligonucleotide microarrays [15]. A third cohort of 130 newly diagnosed patients, with available Affymetrix gene expression data, was used as validation cohort for time to treatment analyses [16]. Clinical characteristics of patients and number and schedules of treatments were previously published [15, 16]. Interphase FISH data of the training cohort were previously published [17]. Affymetrix gene expression data are publicly available via the online Gene Expression Omnibus (http://www.ncbi.nlm.nih.gov/geo/) under accession number GSE22762, GSE39671, and GSE25571. The data were normalized using the robust multichip average (RMA) method [15, 16].

### 2.2. Gene Expression Profiling and Statistical Analyses

The statistical significance of differences in overall survival between groups of patients was calculated by the log-rank test. Multivariate analysis was performed using the Cox proportional hazards model. Survival curves were plotted using the Kaplan-Meier method. All these analyses have been done with R.2.10.1 and bioconductor version 2.5.

### 2.3. Selection of Prognostic Genes on the Training Set

Probe sets were selected for prognostic significance using Maxstat R function (R.2.10.1 and bioconductor version 2.5) and Benjamini Hochberg multiple testing correction [18], yielding 22 significant probe sets in the two independent cohorts of patients with CLL (Table 1).

### 2.4. Validation in the Independent Cohort of Patients

The GE-based risk score of CLL patients was individually calculated and patients were grouped according to the prognostic models and cutoffs from the training cohort. The prognostic value of this scoring was evaluated using log-rank statistics and Cox models.

### 2.5. Gene Set Enrichment Analysis (GSEA)

We compared the gene expression levels from high GE-risk score versus low risk score CLL patients and picked up the genes which had significant different expressions for gene set enrichment analysis (GSEA). Gene set enrichment analysis was carried out by computing overlaps with canonical pathways and gene ontology gene sets obtained from the broad institute [19].

## 3. Results

### 3.1. GE-Based Risk Score in CLL

**Figure 1:**
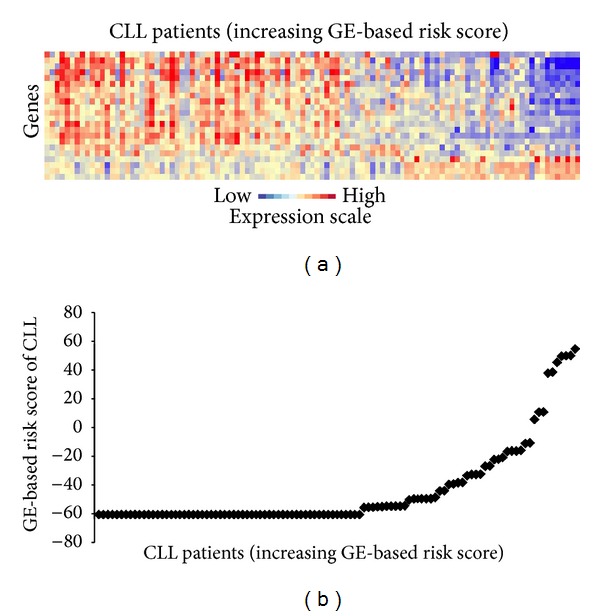
GE-based risk score in CLL patients. (a) Clustergram of genes ordered from best to worst prognosis. The level of the probe set signal is displayed from low (deep blue) to high (deep red) expression. (b) CLL patients (*n* = 107) were ordered by increasing GE-based risk score.

**Figure 2:**
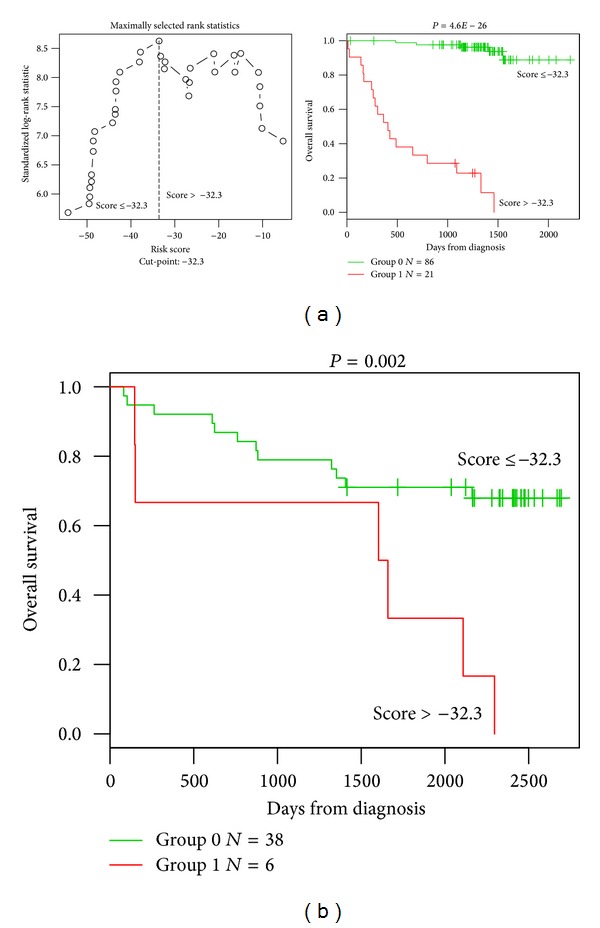
Prognostic value of GE-based risk score in CLL patients. (a) Patients of the training cohort (*n* = 107) were ranked according to increasing GE-based risk score and a maximum difference in OS was obtained with a score = −32.3, splitting patients into a high risk (19,6%) and a low risk (80,4%) groups. (b) The prognostic value of GE-based risk score was assayed on an independent cohort of 44 patients (validation cohort). The parameters to compute GE-based risk score of patients in the validation cohort and the proportions delineating the 2 prognostic groups were those defined with the training cohort.

**Figure 3:**
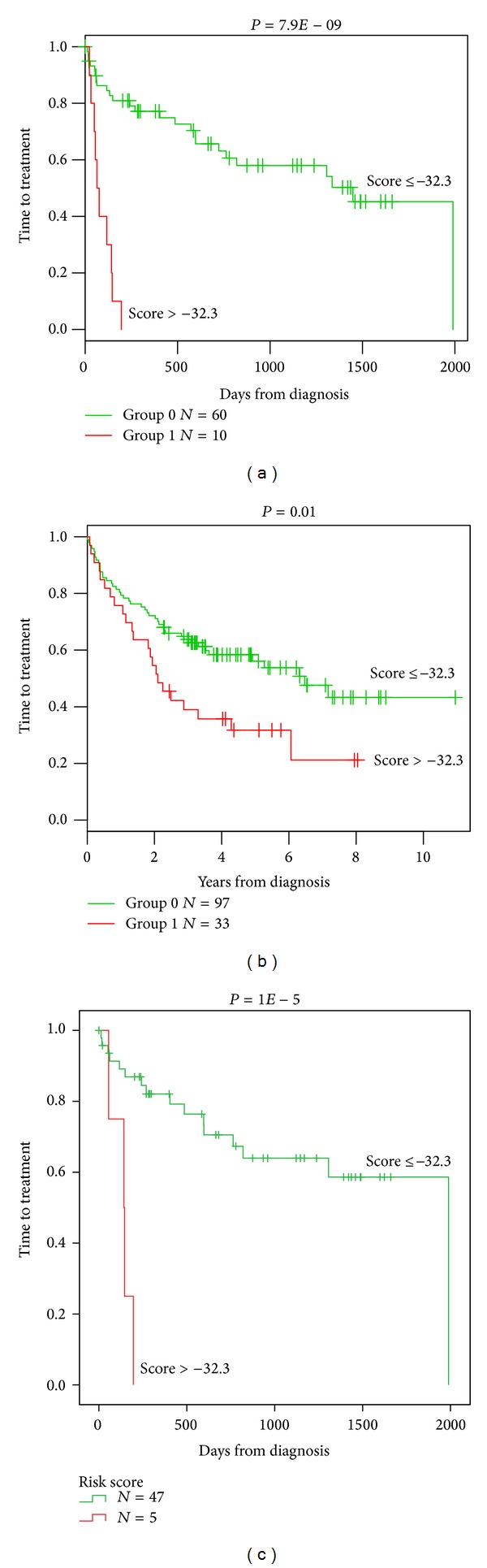
High GE-based risk score is associated with a shorter time to the first treatment in CLL patients. The prognostic value of GE-based risk score was tested in two independent cohorts of CLL patients. A high GE-based risk score is associated with a shorter time to the first treatment in the two independent cohorts ((a) *n* = 70, *P* = 7.9*E* − 9 and (b) *n* = 130, *P* = 0.01) and in patients with cytogenetically defined good prognostic ((c) *n* = 52, *P* = 1*E* − 5).

Using Maxstat R function and Benjamini-Hochberg multiple testing correction [18], 22 probe sets were found to have prognostic value for OS (adjusted *P* value < 0.05) in two independent cohorts of patients with previously-untreated CLL (GSE22762, *n* = 107 and *n* = 44 [15]) (Table 1). These 22 probe sets were probed for 20 unique genes and were used to build a GE-based risk score as reported [20]. Figures 1(a) and 1(b) show expression of the 22 prognostic probe sets and GE-based risk score from patients' tumor samples of the training cohort (ranked according to increasing GE-based risk score). When used as a continuous variable, GE-based risk score had a prognostic value in the two cohorts of patients with CLL (*P* ≤ 10–4, data not shown). Patients of the training cohort (*n* = 107) were ranked according to increased prognostic score and, for a given score value *X*, the difference in survival of patients with a GE-based risk score ≤*X* or >*X* was computed. A maximum difference in overall survival (OS) was obtained with *X* = − 32.3, splitting patients into a high-risk group (19.6% of patients, GE-based risk score > −32.3) with a 13.4 months median OS and a low-risk group (80.4% of patients, GE-based risk score ≤ −32.3) with not reached median survival (Figure 2(a)). The prognostic value of GE-based risk score was validated in an independent CLL patient's cohort (*n* = 44) (Figure 2(b)). Interestingly, a high GE-based risk score is associated with a shorter median time to treatment requirement in two independent cohorts of CLL patients, that is, 2.1 months and 25.2 months for patients with GE score > −32.3 versus 47,7 and 78 months for patients with GE score ≤ −32.3 (*P* = 7.9*E* − 9 and *P* = 0.01, resp.) (Figures 3(a) and 3(b)). In order to investigate the prognostic value of the GE-based risk score in regards to time of first treatment in CLL patients with good prognostic, the analysis was completed in patients without Del17p, without Del11q, and without trisomy 12 known to be associated with a poor prognosis [21]. High GE-based risk score is associated with a shorter time to treatment requirement in patients with cytogenetically defined good prognostic (4.7 months for patients with GE score > −32.3 versus 65.4 for patients with GE score ≤ −32.3, *P* = 1*E* − 5) (Figure 3(c)).

Cox analysis was used to determine whether GE-based risk score provides additional prognostic information compared to previously-identified gene expression-based prognostic markers such as* ADAM29* (a disintegrin and metalloprotease domain 29),* AKAP12* (a kinase (PRKA) anchor protein 12),* DMD*,* LPL*,* NRIP1* (Nuclear receptor-interacting protein 1),* SET10* (Septin 10),* SPG20* (Spastic paraplegia 20),* TCF7*,* TCL1A*,* TPM1* (Tropomyosin 1),* ZAP70* gene expression, the Herold's GEP-based prognostic score (PS8), and Del17p (Table 2) [22–27]. None of these genes were included in the current 20 prognostic genes. Using univariate analyses, GE-based risk score,* ADAM29*,* AKAP12*,* DMD*,* LPL*,* NRIP1*,* SET10*,* SPG20*,* TCF7*,* TCL1A*,* TPM1*,* ZAP70* gene expression, PS8, and Del17p were prognostic (*P* < 0.05, Table 2(a)). When compared two by two, GE-based risk score tested with* NRIP1*,* SPG20*,* TCF7*, and* TPM1* expression, PS8 or Del17p remained significant (*P* < 0.01, Table 2(b)). When all parameters were tested together, only GE-based risk score,* NRIP1,* and* TCF7* gene expression kept prognostic value (Table 2(c)).

Karyotype investigations revealed the association of CLL with del13q14, trisomy 12, del11q22-q23, and del17p13 [1]. Del13q14 is the most frequent alteration that occurs in 50%–60% of cases [1]; trisomy 12 and de11q22-q23 occur in approximately 15% of CLL cases [1] and del17p13 occurs in 5%–10% of untreated CLL patients [28]. Using a cohort of 109 patients with previously untreated CLL (GSE25571) [17], we investigated the association between the GE-based risk score and chromosomal abnormalities. GE-based risk score is significantly higher in patients with del17p13 (Supplementary Figure S1; Supplementary Material is available online at http://dx.doi.org/10.1155/2014/423174).

### 3.2. Combining Prognostic Information of GE-Based Risk Score and* NRIP1* and* TCF7* Expression, into a Single Staging

**Figure 4:**
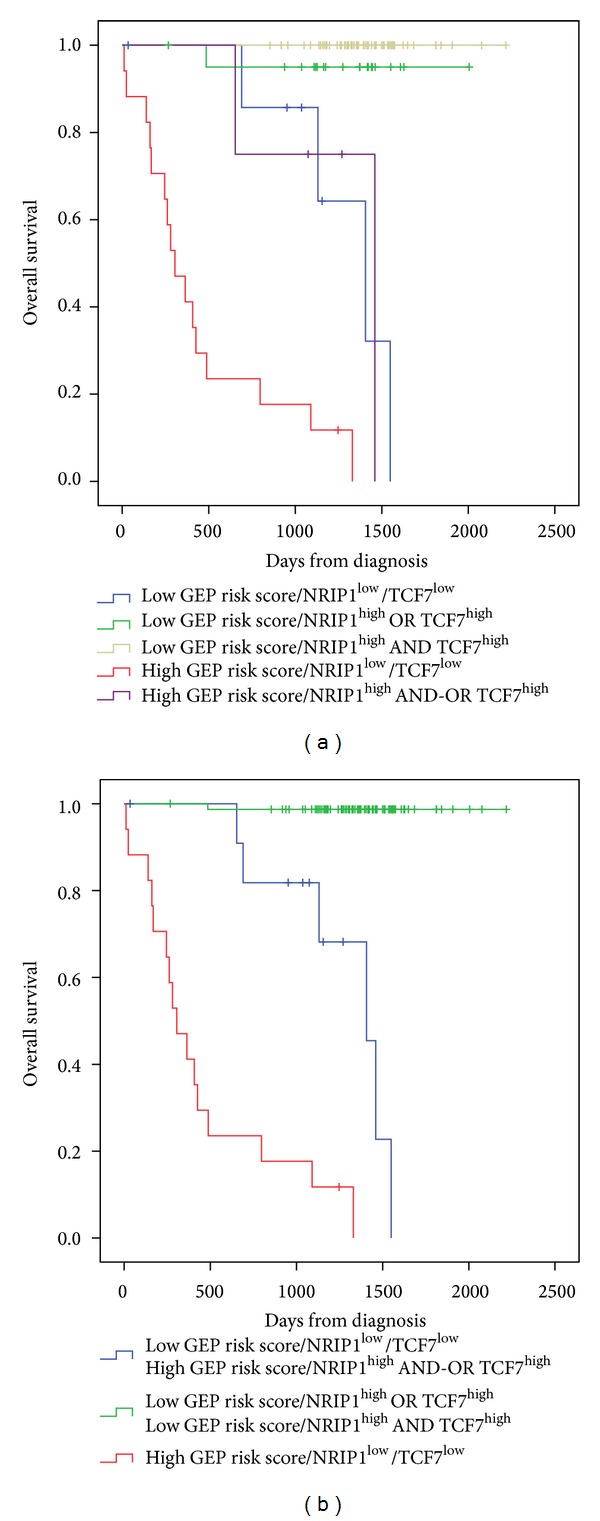
Combination of the prognostic information of GE-based risk score and* NRIP1* and* TCF7* gene expression. (a) Kaplan-Meier analyses were performed to combine the prognostic information of GE-based risk score and* NRIP1* and* TCF7* gene expression. Patients were scored from 1 to 5 according to GE-based risk score in *TCF*7^high  or  Low^ and *NRIP*1^high  or  low^ groups. (b) After merging consecutive groups with no prognostic difference, 3 patient groups with different overall survival (OS) were obtained: I, II, and III (patients of the training cohort, *n* = 107).

Since GE-based risk score and* NRIP1* and* TCF7* expression displayed independent prognostic information, the prognostic information of the GE-based risk score was combined with those of* TCF7* and* NRIP1* gene expression into a single staging. Kaplan-Meier analysis with the 5 patient groups of the training cohort was performed (Figure 4(a)). When 2 consecutive groups showed no prognostic difference, they were merged resulting in a single staging splitting patients into a Group I comprising 72.9% of patients with low GE-based risk score/high* TCF7* or* NRIP1* expression and low GE-based risk score/high* TCF7* and* NRIP1* expression, a Group II comprising 11.2% of patients with low GE-based risk score/low* TCF7*/low* NRIP1* expression and high GE-based risk score/high* TCF7* and-or high* NRIP1* expression, and a Group III comprising 15.9% of patients with high GE-based risk score/low* TCF7*/low* NRIP1* expression (Figure 4(b)). Group I patients had a not reached median OS, patients of groups II and III had, respectively, a median OS of 46.2 months and 10 months (Figure 4(b)).

### 3.3. Tumor Cells Gene Signature in GE-Based High-Risk Group

Gene set enrichment analysis was performed comparing gene expression profiles of CLL patients with high and low GE-based risk score (*n* = 21 and *n* = 86, respectively, in the training cohort). Genes downregulated in CLL patients with mutated IgVH chain compared to unmutated (gene set: FAELT_B_CLL_WITH_VH_REARRANGEMENTS_DN, *P* = 0.001, Supplementary Figure S2 and Table S1) and genes upregulated in CLL patients expressing high levels of lipoprotein lipase (LPL) compared to patients expressing low level of LPL (gene set: BILBAN_B_CLL_LPL_UP, *P* = 0.001, Supplementary Figure S2 and Table S2) were enriched in GE-based high risk group. Conversely, genes involved in chemokine signaling pathway (gene set: PID_CXCR4_PATHWAY, *P* = 0.004, and gene set: KEGG_CHEMOKINE_SIGNALING_PATHWAY, *P* = 0.04, Supplementary Figure S3 and Supplementary Tables S3 and S4) were enriched in GE-based low-risk group.

## 4. Discussion

Following the introduction of microarray methodology in haematological malignancies research, many studies investigated the prediction of reliable prognostic patient subtypes on the basis of their specific gene expression signatures [20, 29, 30]. CLL, although initially reported as an indolent malignancy, is characterized by a highly heterogeneous clinical course, with many patients eventually progressing and requiring therapy [31]. Several large-scale gene expression-based profiling analyses in this malignancy have led to the identification of prognostic factors [11, 13, 22] and development of prognostic signatures for patients' risk stratification [12, 15]. We report here a new GE-based risk score in CLL specimens based on the expression levels of 20 genes documented by 22 probe sets, splitting patients of two independent cohorts into 2 risk categories. None of the 20 genes constituting the GE-based risk score overlap with the previously published prognostic gene signatures for patients' risk stratification [12, 15]. Interestingly, when compared using multivariate analysis, only the current GE-based risk score and* NRIP1* and* TCF7* expression, kept prognostic value.* NRIP1* gene, known as* RIP140*, is a nuclear receptor coregulator with important role in energy homeostasis and a potential involvement in breast cancer [23, 32]. Several reports indicate that* NRIP1* could either inhibit target gene transcription or act as a transcriptional activator.* NRIP1* has been recently described as a novel cell-cycle regulated gene whose expression is directly controlled by E2F transcription factors and increases through their binding to the promoter region [33]. Few studies have analyzed the deregulation of this gene expression in haematological diseases:* NRIP1* has been found to be significantly upregulated in acute myeloid leukemia with complex karyotypes and abnormal chromosome 21 [34]. In CLL,* NRIP1* was shown to be differentially expressed with regard to* IgVH* mutational status [22, 35].

*TCF7* is a member of a family of HMG box containing factors that are known to associate with *β*-catenin in the nucleus to mediate Wnt signaling [36]. The canonical Wnt/*β*-catenin signaling pathway has been shown to play a role in the control of the proliferation, survival, and differentiation of hematopoietic cells [37]. Recent gene expression analyses showed that several members of the Wnt family are overexpressed in CLL cells when compared to their normal counterparts from healthy donors, and this uncontrolled Wnt signaling may contribute to the defect in apoptosis that characterizes this malignancy [38]. The involvement of this pathway in the pathogenesis of several carcinomas, such as colorectal cancer and melanoma, has been also reported [39, 40]. However, there is a significant body of evidence showing that Wnt proteins can function as growth factors for progenitor cells of the B-cell lineage. Indeed, by analyzing the B-cell compartment using LEF1-deficient mice, Reya and colleagues showed a marked reduction of B220^+^ cells in the fetal liver and perinatal bone marrow caused by both increased apoptosis and decreased proliferation [24]. In the same way, an abnormal B-cell development has been observed in mice knocked out for the Wnt receptor Frizzled 9 [25]. In the present study, low expression of TCF7 with high GEP risk score have been correlated with a poor survival. Mice deficient in the TCF7 gene develop intestinal and mammary adenomas, suggesting a role for TCF7 as a tumor suppressor [26]. Furthermore, TCF7 has been also reported to be expressed in hematopoietic stem cells and that its loss diminishes hematopoietic stem/progenitor cell function [27]. These data suggest that the role of Wnt in B-cell malignancies is controversial, as it may have potential oncogenic, as well as tumor suppressor functions. Moreover, Kienle et al. tested the ability of* TCF7* gene to predict the genetic risk in CLL patients, defined by* IgHV* status, V3-21 usage, 11q-, and 17p-.* TCF7* expression provided a high rate of correct assignment of patients at genetic risk [13]. The prognostic impact of our GE-based score associated with* NRIP1* and* TCF7* genes expression should be tested in the context of* IgVH* mutational status, ZAP70 protein expression and* TP53* mutational status.

Among the 20 genes we identified, overexpression of* ERCC1* correlated with a very poor prognosis (HR = 15.0143 and 15.6883 for 203720_s_at and 203719_s_at probes, resp., Table 1). Since many years, it has been shown that treatment of CLL patients with alkylating agents is associated with low rates of complete remission and no improvement in OS [41]. The ability of CLL cells to efficiently repair alkylator-induced DNA damage through DNA repair genes might explain this lack of response. Indeed, ERCC1 forms with Xpf/ERCC4 an endonuclease complex that is involved in nucleotide excision repair (NER) and in repair of drug-induced crosslinks between two complementary strands of DNA, known as interstrand crosslinking (ICL) [42]. For instance, there is evidence that increased expression of ERCC1 in CLL lymphocytes explains the development of resistance to DNA crosslinking agents, for example, nitrogen mustards [43]. In addition, Clingen et al. demonstrated that sensitivity to SJG-136, a highly efficient ICL agent that reacts with guanine bases in a 5′-GATC-3′ sequence in the DNA minor groove, was dependent to some extent on* ERCC1* expression in CHO cells [44]. Fludarabine could enhance the DNA ICL capacity of SJG-136 in primary human CLL cells and thereby offer a rationale for its clinical use in combination with SJG-136 [45]. Furthermore, F11782, a novel dual catalytic inhibitor of topoisomerases I and II, known to be a potent inhibitor of NER could be of therapeutic interest in the GE-based high risk group of CLL patients [46]. More recently, it was demonstrated that a function of PARP in NER DNA repair and clinical grade PARP inhibitors in association with chemotherapy could reverse the resistance of CLL cells to DNA crosslinking agents [47].

Interestingly, GSEA analysis highlighted a significant enrichment of genes downregulated in CLL patients with mutated* IgVH* chain and genes upregulated in CLL patients expressing high levels of lipoprotein lipase in tumor cells of patients within high risk GE-based score group (Supplementary Figure S2 and Supplementary Tables S1 and S2), in particular already known bad prognosis factors* LPL*,* DMD*,* AKAP12,* and* SEPT10* (Supplementary Table S2) [31]. Interestingly, enrichment for IRF4 gene expression was identified in the GE-based high risk group. The t(1,6)(p35.3,p25.2), exclusively found in unmutated CLL, is associated with the involvement of* IRF4 *(Interferon regulatory factor 4) gene. This translocation is observed with high-risk chromosomal aberrations including deletions of 11q and 17p and appears to be associated with an aggressive clinical course [48]. In CLL tumors with low GE-based risk score, GSEA analysis highlighted an enrichment of genes encoding for chemokine signaling pathways (Supplementary Figure S3 and Tables S3 and S4). Of interest, we identified an enrichment of genes involved in the CXCR4 signaling pathway or in the interactions between the CLL tumor cells and their microenvironment (CCL3, CCL4, and CD49d) (Supplementary Table S3). CLL cells express high levels of functional CXCR4 and signaling through this receptor reduces spontaneous and drug-induced apoptosis and also facilitates CLL cell migration beneath stromal cells [49, 50]. More recently, it was demonstrated that the tyrosine kinase inhibitor Dasatinib inhibits CXCR4 signaling in CLL cells and impairs their migration in response to chemokine stimulation [51]. Dasatinib may constitute a potential therapeutic approach in these subgroups of CLL patients. Activated CLL cells secrete CCL3 and CCL4 for the recruitment of immune cells (T cells and monocytes) for cognate interactions. CD49d integrin (VLA-4), expressed on CLL cells, cooperates with chemokine receptors in establishing cell-to-cell adhesion with stromal cells [52]. These data suggested that tumor CLL cells of the GE-based low risk subgroup are more dependent on the interactions with their microenvironment to support their survival and proliferation.

## 5. Conclusion

Given the heterogeneity of CLL patients, the current GE-based risk score combined with* NRIP1* and* TCF7* expression could help in identifying high-risk patients who may benefit from intensive therapeutic strategies and new-targeted treatments.

## Supplementary Material

Supplementary Figure S1: Association between the GE-based risk score and chromosomal abnormalities in CLL patients.
Supplementary Figure S2: Top gene sets significantly associated with high GEP-based risk score.
Supplementary Figure S3: Top gene set significantly associated with low GEP-based risk score.
Supplementary TABLE S1: Genes set enrichment analysis revealed a significant overrepresentation of the gene set FAELT_B_CLL_WITH_VH_REARRANGEMENTS_DN in high risk CLL patients compared to low risk patients (*P*=0.001)
Supplementary TABLE S2: Genes set enrichment analysis revealed a significant overrepresentation of the gene set BILBAN_B_CLL_LPL_UP in high risk CLL patients compared to low risk patients (*P*=0.001).
Supplementary TABLE S3: Genes set enrichment analysis revealed a significant overrepresentation of the gene set PID_CXCR4_PATHWAY in low risk CLL patients compared to high risk patients (*P*=0.004).
Supplementary TABLE S4: Genes set enrichment analysis revealed a significant overrepresentation of the gene set KEGG_CHEMOKINE_SIGNALING_PATHWAY in low risk CLL patients compared to high risk patients (*P*=0.04).

## Figures and Tables

**Table 1:** List of the 22 probe sets associated with a prognostic value in CLL patients.

Probe set	Gene symbol	Gene name	Hazard Ratio
218559_s_at	MAFB	v-maf musculoaponeurotic fibrosarcoma oncogene homolog B	0.0471
203413_at	NELL2	NEL-like 2	0.0481
212761_at	TCF7L2	transcription factor 7-like 2	0.0497
206157_at	PTX3	pentraxin 3, long	0.0537
219947_at	CLEC4A	C-type lectin domain family 4, member A	0.0566
209871_s_at	APBA2	amyloid beta precursor protein-binding, family A, member 2	0.0568
204526_s_at	TBC1D8	TBC1 domain family, member 8	0.063
218793_s_at	SCML1	sex comb on midleg-like 1	0.0636
225924_at	FNIP2	folliculin interacting protein 2	0.0638
207075_at	NLRP3	NLR family, pyrin domain containing 3	0.0663
221698_s_at	CLEC7A	C-type lectin domain family 7, member A	0.0667
226876_at	FAM101B	family with sequence similarity 101, member B	0.0691
212239_at	PIK3R1	phosphoinositide-3-kinase, regulatory subunit 1 (alpha)	0.0731
203397_s_at	GALNT3	UDP-N-acetyl-alpha-D-galactosamine:polypeptide N-acetylgalactosaminyltransferase 3	0.0739
216037_x_at	TCF7L2	transcription factor 7-like 2	0.0781
226279_at	PRSS23	protease, serine, 23	0.0833
229699_at	LOC100129550	hypothetical LOC100129550	0.0841
244598_at	LCP2	lymphocyte cytosolic protein 2	0.0907
209183_s_at	C10orf10	chromosome 10 open reading frame 10	14.5055
203720_s_at	ERCC1	excision repair cross-complementing rodent repair deficiency, complementation group 1	15.0143
203719_at	ERCC1	excision repair cross-complementing rodent repair deficiency, complementation group 1	15.6883
221725_at	WASF2	WAS protein family, member 2	16.2394

Hazard ratios (HR) are indicated for each gene used to design GE-based risk score in CLL patients. Probe sets are sorted by increasing HR.

**Table:** (a)

Prognostic variable	Overall survival	
	(*n* = 107)	
	HR	*P* value
GEP risk score	45.39	<0.0001
* ADAM29 *	0.40	0.04
* AKAP12 *	2.60	0.02
* DMD *	3.39	0.004
* LPL *	4.19	0.001
* NRIP1 *	0.12	<0.0001
* SEPT10 *	2.95	0.01
* SPG20 *	0.28	0.006
* TCF7 *	0.35	<0.0001
* TCL1A *	4.14	0.001
* TPM1 *	2.50	0.02
* ZAP70 *	3.28	0.02
* PS8 *	10.40	<0.0001
Del(17p)	10.13	<0.0001

**Table:** (b)

Prognostic variables compared two by two	Overall survival	
	(*n* = 107)	
	HR	*P* value
GEP risk score	55.30	<0.0001
*ADAM29 *	1.63	NS

GEP risk score	46.71	<0.0001
*AKAP12 *	0.93	NS

GEP risk score	51.78	<0.0001
*DMD *	0.75	NS

GEP risk score	40.52	<0.0001
*LPL *	1.49	NS

GEP risk score	8.29	<0.0001
*NRIP1 *	0.035	0.003

GEP risk score	50.95	<0.0001
*SEPT10 *	0.76	NS

GEP risk score	53.31	<0.0001
*SPG20 *	0.20	0.001

GEP risk score	28.34	<0.0001
*TCF7 *	0.067	0.001

GEP risk score	40.39	<0.0001
*TCL1A *	2.33	NS

GEP risk score	69.60	<0.0001
*TPM1 *	4.77	0.001

GEP risk score	44.60	<0.0001
*ZAP70 *	1.04	NS

GEP risk score	10.35	<0.0001
*PS8 *	4.73	0.001

GEP risk score	38.95	<0.0001
Del(17p)	3.14	0.02

**Table:** (c)

All prognostic variables	Overall survival	
	(*n* = 107)	
	HR	*P* value
GEP risk score	26.23	0.002
* ADAM29 *	1.04	NS
* AKAP12 *	6.89	NS
* DMD *	0.58	NS
* LPL *	0.54	NS
* NRIP1 *	0.017	0.01
* SEPT10 *	0.58	NS
* SPG20 *	0.76	NS
* TCF7 *	0.022	0.01
* TCL1A *	0.29	NS
* TPM1 *	2.83	NS
* ZAP70 *	1.53	NS
* PS8 *	0.63	NS
Del(17p)	1.94	NS

The prognostic factors were tested as single variable (a) or multivariables (b, c) using Cox-model. *P* values and the hazard ratios (HR) are shown. NS: not significant at a 5% threshold.
