# Supplementary material for: Identification of a 20-Gene Expression-Based Risk Score as a Predictor of Clinical Outcome in Chronic Lymphocytic Leukemia Patients
Source: Biomed Res Int. 2014 May 5;2014:423174. doi: 10.1155/2014/423174 (PMC4026849; doi:10.1155/2014/423174)
Supplement: Supplementary file 1 — Supplementary Figure S1: Association between the GE-based risk score and chromosomal abnormalities in CLL patients. Supplementary Figure S2: Top gene sets significantly associated with high GEP-based risk score. Supplementary Figure S3: Top gene set significantly associated with low GEP-based risk score. Supplementary TABLE S1: Genes set enrichment analysis revealed a significant overrepresentation of the gene set FAELT_B_CLL_WITH_VH_REARRANGEMENTS_DN in high risk CLL patients compared to low risk patients (P=0.001) Supplementary TABLE S2: Genes set enrichment analysis revealed a significant overrepresentation of the gene set BILBAN_B_CLL_LPL_UP in high risk CLL patients compared to low risk patients (P=0.001). Supplementary TABLE S3: Genes set enrichment analysis revealed a significant overrepresentation of the gene set PID_CXCR4_PATHWAY in low risk CLL patients compared to high risk patients (P=0.004). Supplementary TABLE S4: Genes set enrichment analysis revealed a significant overrepresentation of the gene set KEGG_CHEMOKINE_SIGNALING_PATHWAY in low risk CLL patients compared to high risk patients (P=0.04). [file 423174.f1.pdf]

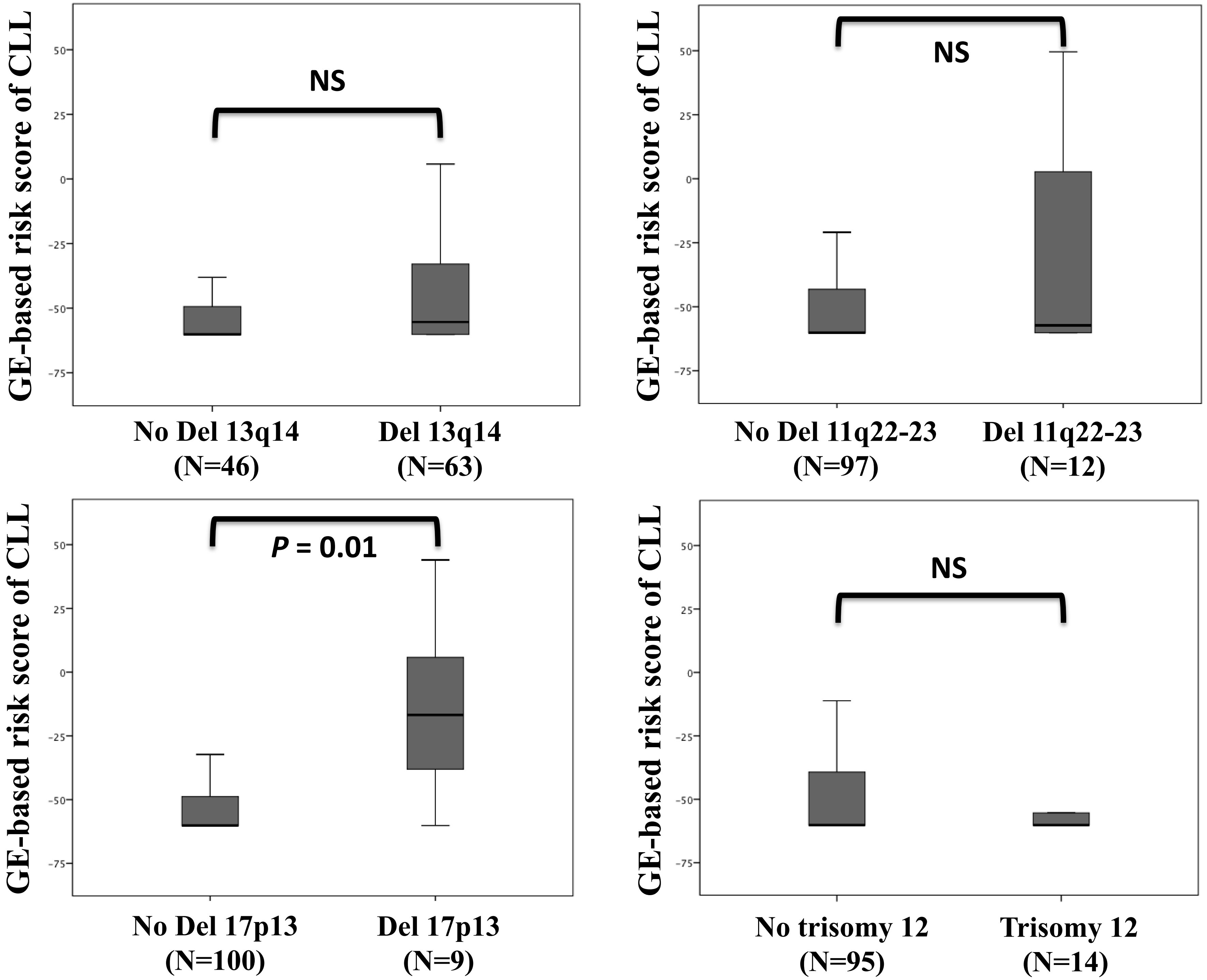
GE-based risk score of CLL
50
25
0
-25
-50
-75
NS
No Del 13q14 (N=46)
Del 13q14 (N=63)
NS
No Del 11q22-23 (N=97)
Del 11q22-23 (N=12)
P = 0.01
No Del 17p13 (N=100)
Del 17p13 (N=9)
NS
No trisomy 12 (N=95)
Trisomy 12 (N=14)

Supplementary Figure S1. GEP-based risk score.

Association between the GE-based risk score and chromosomal abnormalities in CLL patients.

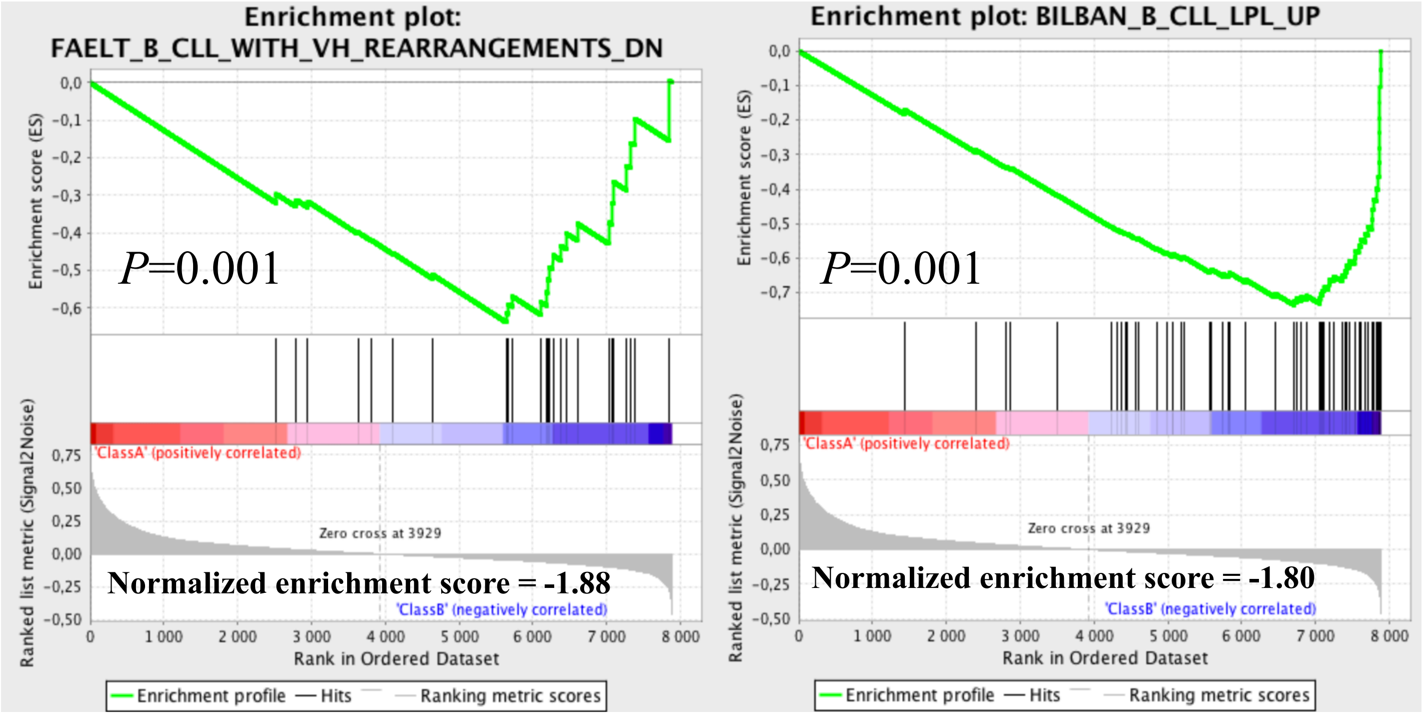

Supplementary Figure S2. Top gene sets significantly associated with high GEP-based risk score.

GSEA enrichment plots with the absolute enrichment p value and the normalized enrichment score of the gene set.

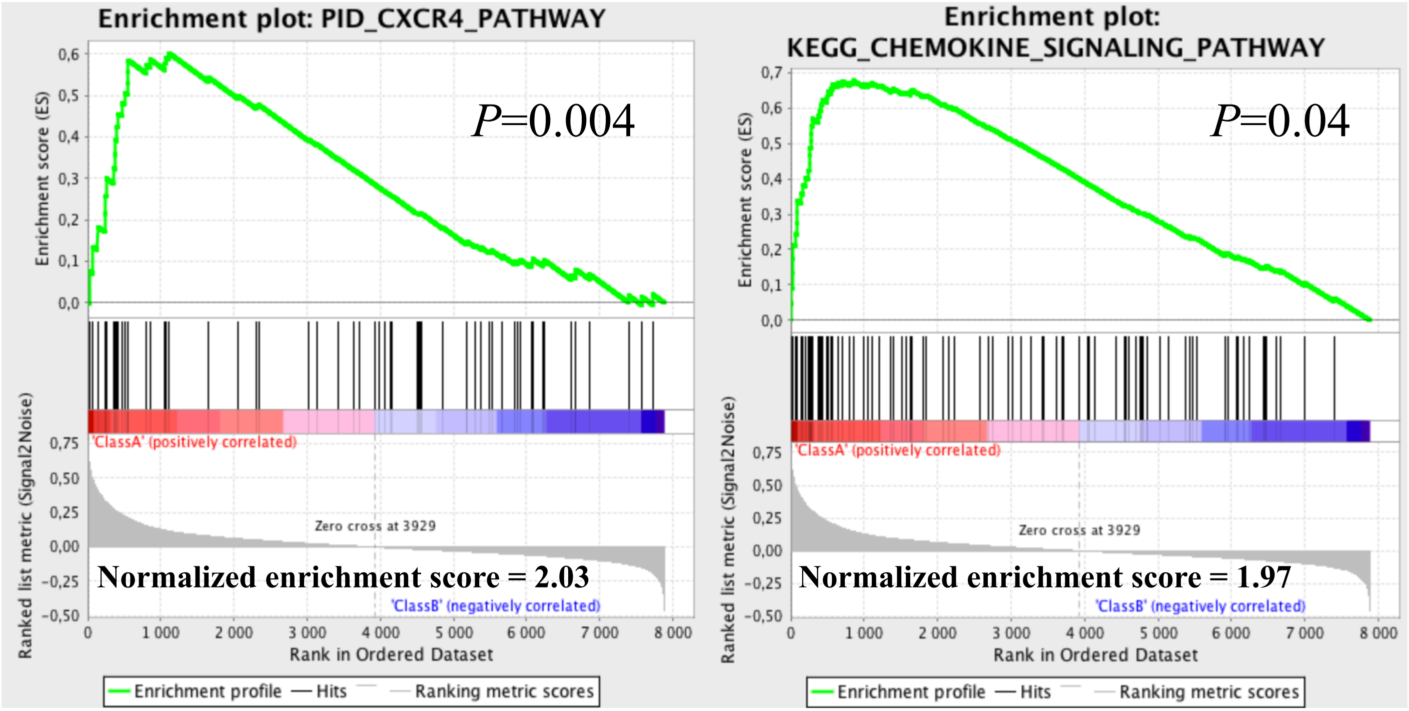

Supplementary Figure S3. Top gene set significantly associated with low GEP-based risk score.

GSEA enrichment plot with the absolute enrichment p value and the normalized enrichment score of the gene set.

Supplementary TABLE S1: Genes set enrichment analysis revealed a significant overrepresentation of the gene set FAELT_B_CLL_WITH_VH_REARRANGEMENTS_DN in high risk CLL patients compared to low risk patients (*P*=*0*.001).

PROBE	GENE SYMBOL	GENE_TITLE	RANK METRIC SCORE	RUNNING Enrichment Score	CORE ENRICHMENT
XBP1	XBP1	X-box binding protein 1	-0.04499693214893341	-0.6130199	Yes
VPS13B	VPS13B	vacuolar protein sorting 13B (yeast)	-0.045701898634433746	-0.5900873	Yes
ZHX2	ZHX2	zinc fingers and homeoboxes 2	-0.047306593507528305	-0.5700497	Yes
PARP4	PARP4	poly (ADP-ribose) polymerase family, member 4	-0.05936216562986374	-0.5849426	Yes
UBE2D2	UBE2D2	ubiquitin-conjugating enzyme E2D 2 (UBC4/5 homolog, yeast)	-0.06224088743329048	-0.5583657	Yes
CBX7	CBX7	chromobox homolog 7	-0.062492795288562775	-0.5240116	Yes
HSPA4	HSPA4	heat shock 70kDa protein 4	-0.06306540966033936	-0.49072808	Yes
PBX3	PBX3	pre-B-cell leukemia transcription factor 3	-0.06499937176704407	-0.45976883	Yes
ASMTL	ASMTL	acetylserotonin O-methyltransferase-like	-0.06841114908456802	-0.43384722	Yes
TMEM147	TMEM147	transmembrane protein 147	-0.07219166308641434	-0.4018115	Yes
PFN2	PFN2	profilin 2	-0.07871776074171066	-0.37531516	Yes
MRPL40	MRPL40	mitochondrial ribosomal protein L40	-0.09883939474821091	-0.3726303	Yes
PFKP	PFKP	phosphofructokinase, platelet	-0.10060647130012512	-0.3199549	Yes
GLO1	GLO1	glyoxalase I	-0.10182798653841019	-0.26479724	Yes
MAGED2	MAGED2	melanoma antigen family D, 2	-0.1134805828332901	-0.22126743	Yes
SLC35D2	SLC35D2	solute carrier family 35, member D2	-0.11825907230377197	-0.16011041	Yes
MYLK	MYLK	myosin, light chain kinase	-0.1252080351114273	-0.09559967	Yes
LDOC1	LDOC1	leucine zipper, down-regulated in cancer 1	-0.2783927023410797	0.004070683	Yes

Supplementary TABLE S2: Genes set enrichment analysis revealed a significant overrepresentation of the gene set BILBAN_B_CLL_LPL_UP in high risk CLL patients compared to low risk patients (*P=0*.001).

PROBE	GENE SYMBOL	GENE_TITLE	RANK METRIC SCORE	RUNNING Enrichment Score	CORE ENRICHMENT
TBC1D1	TBC1D1	TBC1 (tre-2/USP6, BUB2, cdc16) domain family, member 1	-0.08338106423616409	-0.7253143	Yes
CPT1A	CPT1A	carnitine palmitoyltransferase 1A (liver)	-0.08443442732095718	-0.7172482	Yes
SLC39A14	SLC39A14	solute carrier family 39 (zinc transporter), member 14	-0.0874563530087471	-0.71323246	Yes
IRF4	IRF4	interferon regulatory factor 4	-0.09082043170928955	-0.7096421	Yes
PNMA2	PNMA2	paraneoplastic antigen MA2	-0.09997770935297012	-0.7176791	Yes
CEACAM1	CEACAM1	carcinoembryonic antigen-related cell adhesion molecule 1	-0.10055960714817047	-0.7050652	Yes
MYBL2	MYBL2	v-myb myeloblastosis viral oncogene homolog (avian)-like 2	-0.1016419380903244	-0.69408906	Yes
AZIN1	AZIN1	antizyme inhibitor 1	-0.10350252687931061	-0.68323684	Yes
TMEM140	TMEM140	transmembrane protein 140	-0.10717408359050751	-0.6757056	Yes
DNMBP	DNMBP	dynamin binding protein	-0.10790828615427017	-0.6621949	Yes
SLC23A2	SLC23A2	solute carrier family 23 (nucleobase transporters), member 2	-0.11094300448894501	-0.65298843	Yes
RRBP1	RRBP1	ribosome binding protein 1 homolog 180kDa (dog)	-0.12251496315002441	-0.65060097	Yes
ST6GALNAC4	ST6GALNAC4	ST6-N-acetylgalactosaminide alpha-2,6-sialyltransferase 4	-0.12699449062347412	-0.637879	Yes
DIP2C	DIP2C	DIP2 disco-interacting protein 2 homolog C (Drosophila)	-0.12917253375053406	-0.6225537	Yes
WSB2	WSB2	WD repeat and SOCS box-containing 2	-0.13311201333999634	-0.6091067	Yes
IGSF3	IGSF3	immunoglobulin superfamily, member 3	-0.14086134731769562	-0.59790117	Yes
IGHD	IGHD	immunoglobulin heavy constant delta	-0.14238741993904114	-0.57945585	Yes
CREB3L2	CREB3L2	cAMP responsive element binding protein 3-like 2	-0.1480318158864975	-0.5645676	Yes
FCER2	FCER2	Fc fragment of IgE, low affinity II, receptor for (CD23)	-0.1534823179244995	-0.54674757	Yes
ATOX1	ATOX1	ATX1 antioxidant protein 1 homolog (yeast)	-0.168036550283432	-0.52996475	Yes
AKAP12	AKAP12	A kinase (PRKA) anchor protein (gravin) 12	-0.1812814623117447	-0.5100578	Yes
ABCA6	ABCA6	ATP-binding cassette, sub-family A (ABC1), member 6	-0.20973779261112213	-0.48695025	Yes
FRY	FRY	furry homolog (Drosophila)	-0.21455228328704834	-0.45806098	Yes
BCL7A	BCL7A	B-cell CLL/lymphoma 7A	-0.22043849527835846	-0.42898992	Yes
SGCE	SGCE	sarcoglycan, epsilon	-0.24693357944488525	-0.3992914	Yes
ARMCX2	ARMCX2	armadillo repeat containing, X-linked 2	-0.27560511231422424	-0.36457357	Yes
ANKRD57	ANKRD57	ankyrin repeat domain 57	-0.2953161895275116	-0.32429698	Yes
KLK2	KLK2	kallikrein 2, prostatic	-0.3105448782444	-0.2816418	Yes
DFNA5	DFNA5	deafness, autosomal dominant 5	-0.3124789595603943	-0.2383337	Yes
SEPT10	SEPT10	septin 10	-0.3144703805446625	-0.1946202	Yes
PEG10	PEG10	paternally expressed 10	-0.3283138573169708	-0.14897676	Yes
PHF16	PHF16	PHD finger protein 16	-0.33501136302948	-0.10227182	Yes
DMD	DMD	dystrophin (muscular dystrophy, Duchenne and Becker types)	-0.3533475995063782	-0.053138327	Yes
LPL	LPL	lipoprotein lipase	-0.389405220746994	6.3877E-4	Yes

Supplementary TABLE S3: Genes set enrichment analysis revealed a significant overrepresentation of the gene set PID_CXCR4_PATHWAY in low risk CLL patients compared to high risk patients (*P*=*0*.004).

PROBE	GENE SYMBOL	GENE_TITLE	RANK METRIC SCORE	RUNNING ES	CORE ENRICHMENT
PAG1	PAG1	phosphoprotein associated with glycosphingolipid microdomains 1	0.597561240196228	0.0776465	Yes
CD247	CD247	CD247 molecule	0.4821421802043915	0.13548093	Yes
CD3G	CD3G	CD3g molecule, gamma (CD3-TCR complex)	0.41322290897369385	0.18331309	Yes
PIK3R5	PIK3R5	phosphoinositide-3-kinase, regulatory subunit 5, p101	0.33174756169319153	0.21498702	Yes
ITGA4	ITGA4	integrin, alpha 4 (antigen CD49D, alpha 4 subunit of VLA-4 receptor)	0.3287671208381653	0.2582759	Yes
CD3D	CD3D	CD3d molecule, delta (CD3-TCR complex)	0.32326313853263855	0.30018815	Yes
CD4	CD4	CD4 molecule	0.27912190556526184	0.325577	Yes
PLCB1	PLCB1	phospholipase C, beta 1 (phosphoinositide-specific)	0.2644299268722534	0.35768825	Yes
FYN	FYN	FYN oncogene related to SRC, FGR, YES	0.2613527774810791	0.39207122	Yes
PIK3R1	PIK3R1	phosphoinositide-3-kinase, regulatory subunit 1 (p85 alpha)	0.2568548321723938	0.42482895	Yes
CD3E	CD3E	CD3e molecule, epsilon (CD3-TCR complex)	0.24974220991134644	0.4557389	Yes
ITGA6	ITGA6	integrin, alpha 6	0.23540905117988586	0.4810216	Yes
HCK	HCK	hemopoietic cell kinase	0.22374702990055084	0.50665873	Yes
ITGA5	ITGA5	integrin, alpha 5 (fibronectin receptor, alpha polypeptide)	0.21087005734443665	0.52967566	Yes
PTK2	PTK2	PTK2 protein tyrosine kinase 2	0.2093726545572281	0.55696535	Yes
YES1	YES1	v-yes-1 Yamaguchi sarcoma viral oncogene homolog 1	0.2083330899477005	0.5844991	Yes
PXN	PXN	paxillin	0.15401707589626312	0.57305706	Yes
GNAI3	GNAI3	guanine nucleotide binding protein (G protein), alpha inhibiting activity polypeptide 3	0.1462269425392151	0.5872838	Yes
ARRB2	ARRB2	arrestin, beta 2	0.12091217190027237	0.57715714	Yes
MMP9	MMP9	matrix metallopeptidase 9 (gelatinase B, 92kDa gelatinase, 92kDa type IV collagenase)	0.11903076618909836	0.5915739	Yes
PIK3CB	PIK3CB	phosphoinositide-3-kinase, catalytic, beta polypeptide	0.11373784393072128	0.602086	Yes

Supplementary TABLE S4: Genes set enrichment analysis revealed a significant overrepresentation of the gene set KEGG_CHEMOKINE_SIGNALING_PATHWAY in low risk CLL patients compared to high risk patients (*P*=*0*.04).

PROBE	GENE SYMBOL	GENE_TITLE	RANK METRIC SCORE	RUNNING Enrichment Score	CORE ENRICHMENT
CXCL2	CXCL2	chemokine (C-X-C motif) ligand 2	0.6935211420059204	0.04809883	Yes
TIAM1	TIAM1	T-cell lymphoma invasion and metastasis 1	0.6424120664596558	0.09198278	Yes
CCR1	CCR1	chemokine (C-C motif) receptor 1	0.5966988801956177	0.13279931	Yes
CCL20	CCL20	chemokine (C-C motif) ligand 20	0.592612087726593	0.17410034	Yes
CXCL3	CXCL3	chemokine (C-X-C motif) ligand 3	0.5661141276359558	0.21200848	Yes
ITK	ITK	IL2-inducible T-cell kinase	0.49852678179740906	0.24403626	Yes
XCL2	XCL2	chemokine (C motif) ligand 2	0.46367451548576355	0.2732424	Yes
CXCL1	CXCL1	chemokine (C-X-C motif) ligand 1 (melanoma growth stimulating activity, alpha)	0.45889031887054443	0.30493826	Yes
IL8	IL8	interleukin 8	0.45861756801605225	0.3370001	Yes
GNG11	GNG11	guanine nucleotide binding protein (G protein), gamma 11	0.4110870659351349	0.3599625	Yes
CCL2	CCL2	chemokine (C-C motif) ligand 2	0.3784455358982086	0.38218337	Yes
CXCL5	CXCL5	chemokine (C-X-C motif) ligand 5	0.36029547452926636	0.40416232	Yes
PIK3R5	PIK3R5	phosphoinositide-3-kinase, regulatory subunit 5, p101	0.33174756169319153	0.42260504	Yes
CCL5	CCL5	chemokine (C-C motif) ligand 5	0.3235359787940979	0.44316947	Yes
CX3CR1	CX3CR1	chemokine (C-X3-C motif) receptor 1	0.3233361840248108	0.46577385	Yes
PPBP	PPBP	pro-platelet basic protein (chemokine (C-X-C motif) ligand 7)	0.3223371207714081	0.48805162	Yes
PF4	PF4	platelet factor 4 (chemokine (C-X-C motif) ligand 4)	0.30851754546165466	0.50666755	Yes
CCL4	CCL4	chemokine (C-C motif) ligand 4	0.306867778301239	0.52786386	Yes
PRKACB	PRKACB	protein kinase, cAMP-dependent, catalytic, beta	0.30548328161239624	0.54883504	Yes
CCL7	CCL7	chemokine (C-C motif) ligand 7	0.30158886313438416	0.5692772	Yes
PLCB1	PLCB1	phospholipase C, beta 1 (phosphoinositide-specific)	0.2644299268722534	0.5772371	Yes
PIK3R1	PIK3R1	phosphoinositide-3-kinase, regulatory subunit 1 (p85 alpha)	0.2568548321723938	0.5927548	Yes
PF4V1	PF4V1	platelet factor 4 variant 1	0.25288277864456177	0.6090217	Yes
CXCL10	CXCL10	chemokine (C-X-C motif) ligand 10	0.24867390096187592	0.6243526	Yes
CXCL16	CXCL16	chemokine (C-X-C motif) ligand 16	0.22437630593776703	0.6307961	Yes
HCK	HCK	hemopoietic cell kinase	0.22374702990055084	0.6460531	Yes
PTK2	PTK2	PTK2 protein tyrosine kinase 2	0.2093726545572281	0.6545285	Yes
ARRB1	ARRB1	arrestin, beta 1	0.20537954568862915	0.6661908	Yes
GNG8	GNG8	guanine nucleotide binding protein (G protein), gamma 8	0.18877199292182922	0.67117214	Yes
CCL28	CCL28	chemokine (C-C motif) ligand 28	0.17370964586734772	0.6749721	Yes
PXN	PXN	paxillin	0.15401707589626312	0.67264575	Yes
GNAI3	GNAI3	guanine nucleotide binding protein (G protein), alpha inhibiting activity polypeptide 3	0.1462269425392151	0.6774769	Yes
